# Supplementary material for: Identification of osteogenic progenitor cell-targeted peptides that augment bone formation
Source: Nat Commun. 2020 Aug 27;11:4278. doi: 10.1038/s41467-020-17417-9 (PMC7453024; doi:10.1038/s41467-020-17417-9)
Supplement: Supplementary file 1 — Supplementary Information [file 41467_2020_17417_MOESM1_ESM.docx]

**Supplementary Figure 1**. The synthetic approach of focused OBOC library


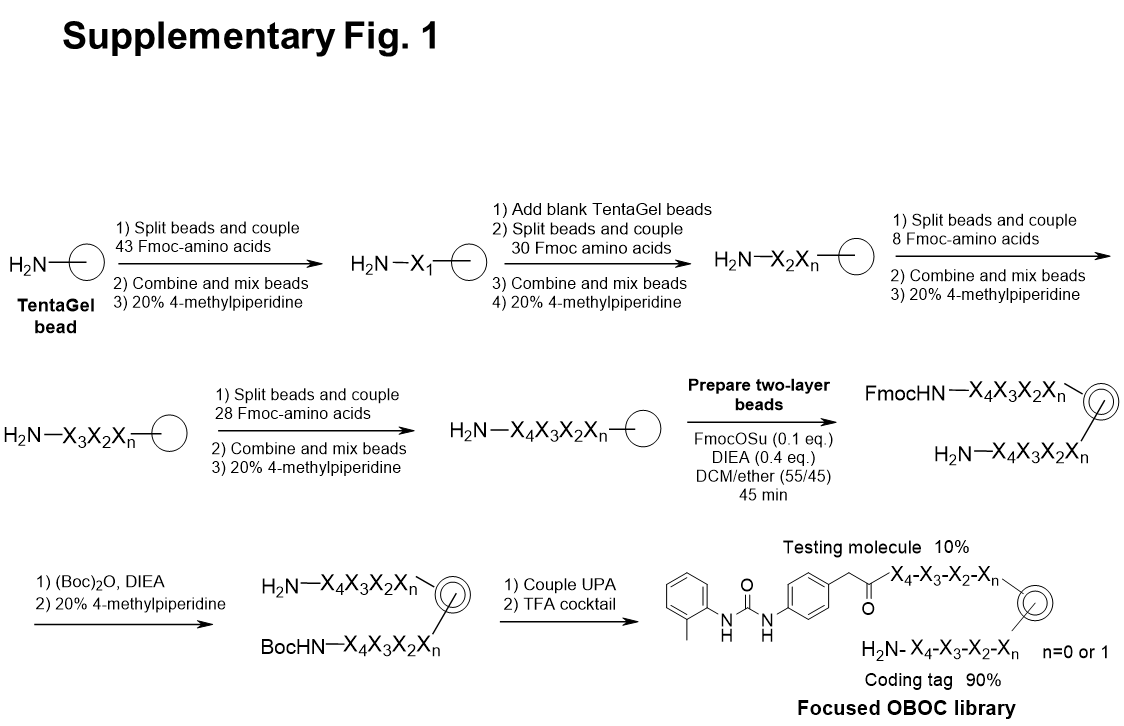


**Supplementary Figure 2**.

Lymphocyte binding. (a) ALP levels of select peptides cultured with mouse MSC for 7 and 14 days. (b) Peripheral mononuclear cells were extracted from the blood and incubated with the beads displaying scramble, YLL3, or YLL8 peptides for 1 hour. Columns represented individual values in each group and the error bars represented standard deviations. N=6/group.*, p < 0.05 using one-way ANOVA followed by Dunnett’s multiple comparison post hoc test for multiple comparisons between the PBS control group. Scale Bar =100µm.


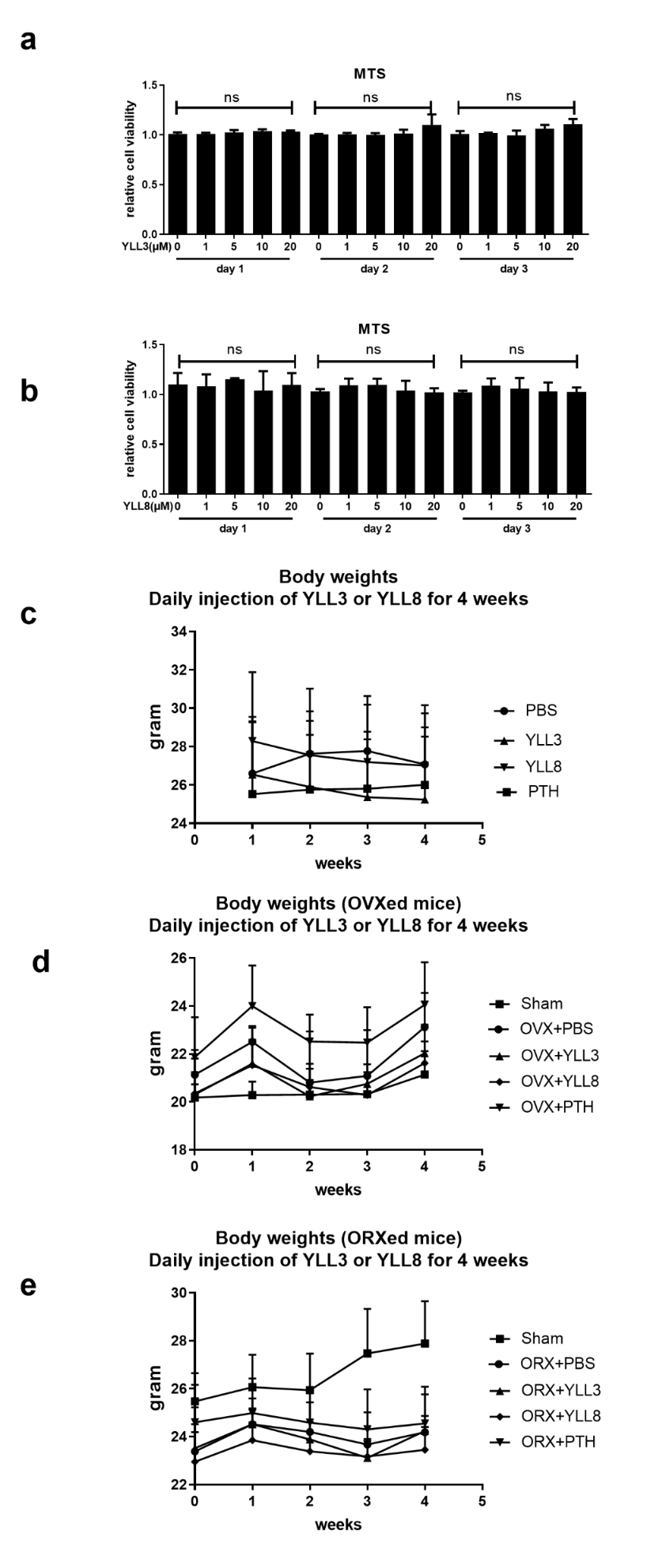

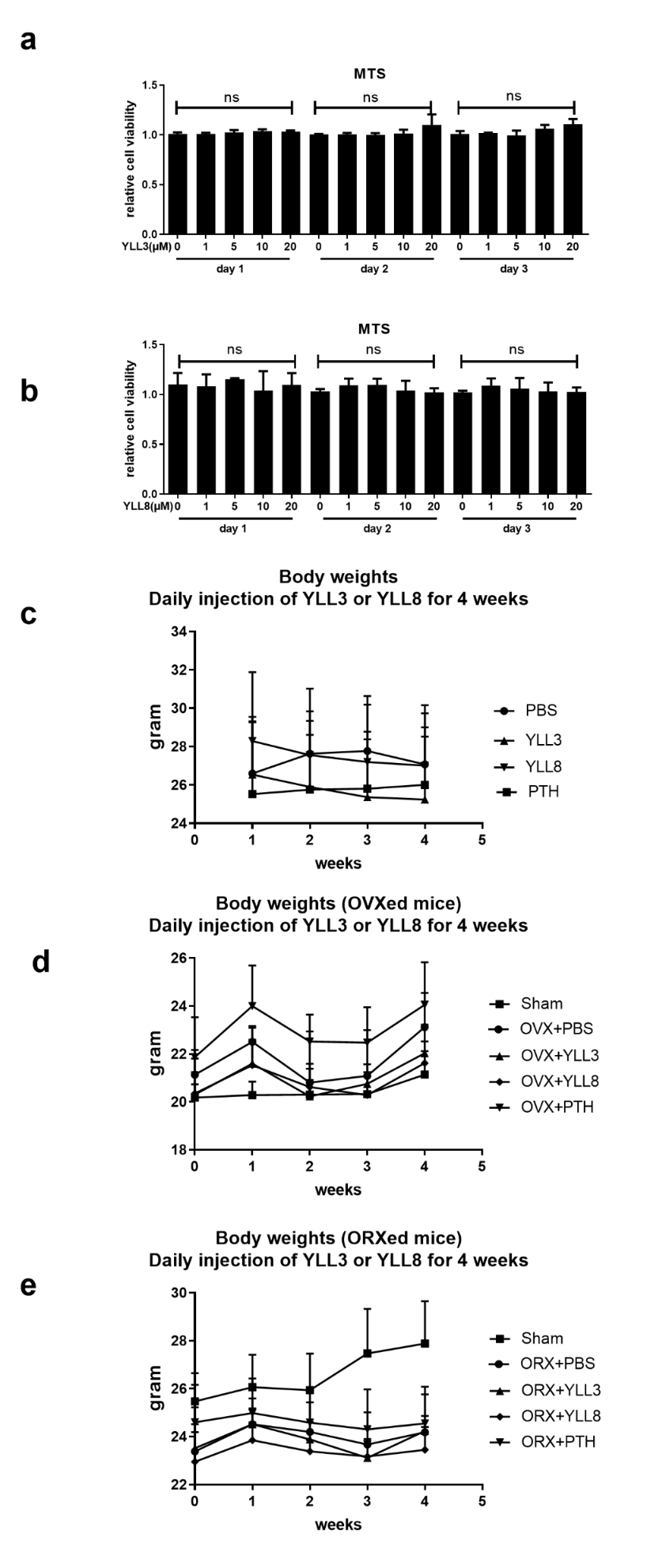


**Supplementary Figure 3**. (a) MTS results for YLL3 and (b) for YLL8 after cultured with human MSC for up to three days in indicated concentrations. (c) Bodyweight changes in the experiment when YLL3 and YLL8 were given daily to intact adult female mice for 4 weeks. (d) Bodyweight changes in the experiment when YLL3 and YLL8 were given daily to ovariectomized mice for four weeks. (**e**) Bodyweight changes in the experiment when YLL3 and YLL8 were given daily to orchiectomized mice for four weeks. N=6 animal/group. Mean +/- SD was presented.


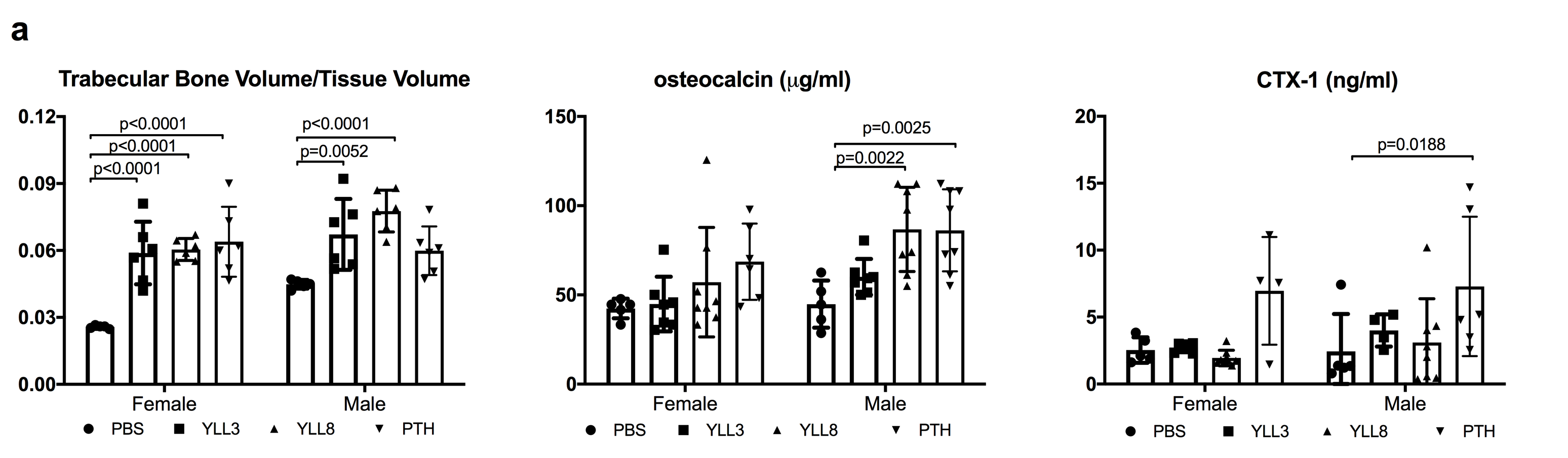

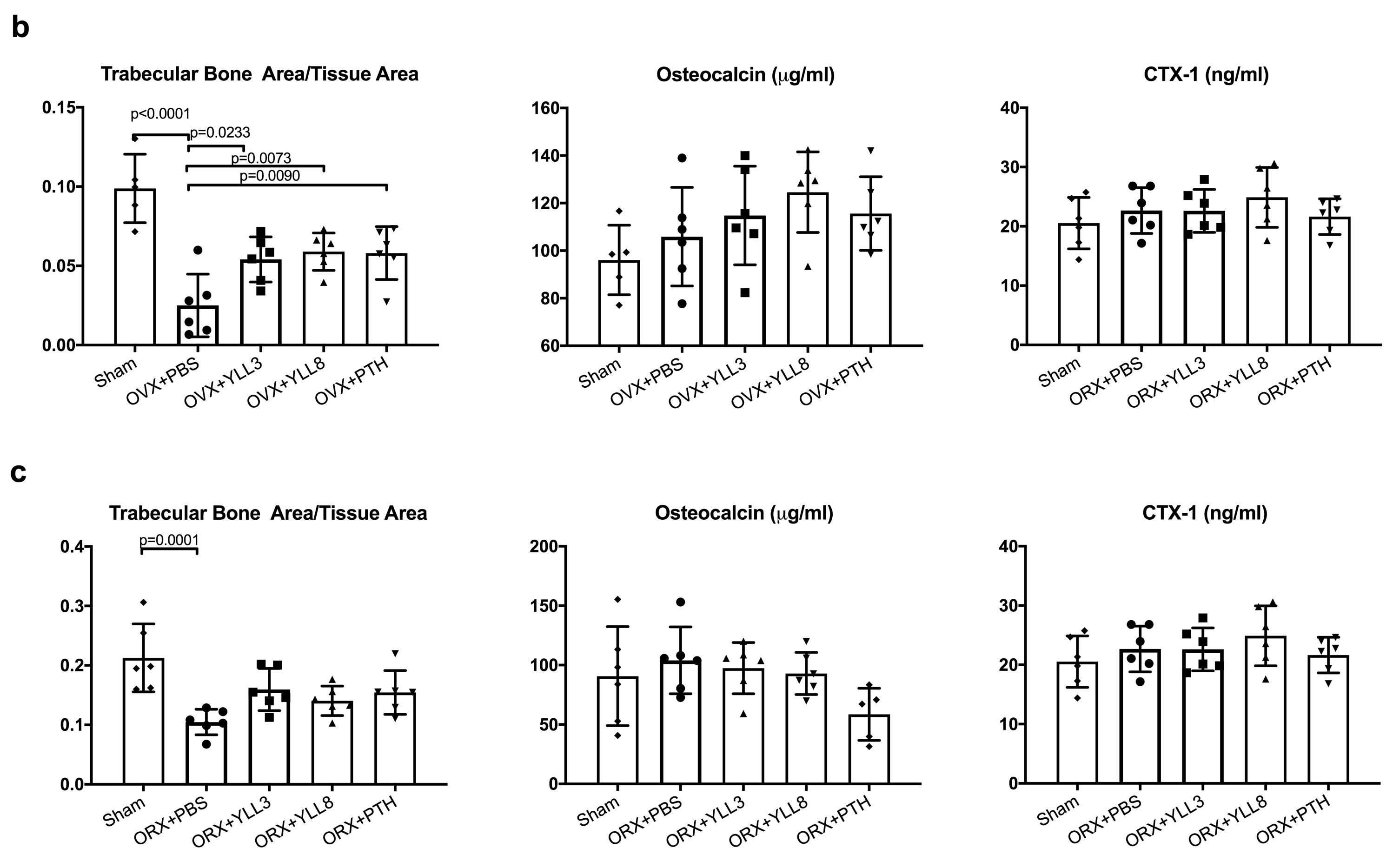


**Supplementary Figure 4**. Bone mass and serum bone turnover markers in the *in vivo* studies. (a) 4-month-old female mice were treated with PBS control, YLL3, or YLL8 at 10 μg/kg, or hPTH(-134) at 25 μg/kg, sc., 5x/week for 28 days. Trabecular bone volume was obtained by microCT scans at the distal femoral metaphysis. Osteocalcin and CTX-1 were measured at the serum. (b) 2-month-old female mice were ovariectomized and treated with PBS, YLL3 or YLL8 at 10 μg/kg or hPTH (1-34) at 40 μg/kg, sc., 5x/week for 28 days. The trabecular bone area was measured by bone histomorphometry at the proximal tibiae. Osteocalcin and CTX-1 were measured at the serum. (**c**) 2-month-old male mice were orchiectomized and treated with PBS, YLL3, or YLL8 at 10 μg/kg or hPTH (1-34) at 40 μg/kg, sc., 5x/week for 28 days. The trabecular bone area was measured by bone histomorphometry at the 5^th^ lumbar vertebral bodies. Osteocalcin and CTX-1 were measured at the serum. Columns represented individual values in each group and the error bars represented standard deviations. N≥6 animals/group. P values were derived from one-way ANOVA followed by Dunnett’s multiple comparison post hoc test for comparisons between the treated groups verse vehicle control within the same sex.

**Supplementary Figure 5**. Expressions of Prx1 and osterix in the bone. Representative distal femurs were shown for Prx1-GFP (green) and osterix-mCherry (red) mice. Scale Bar =100µm.


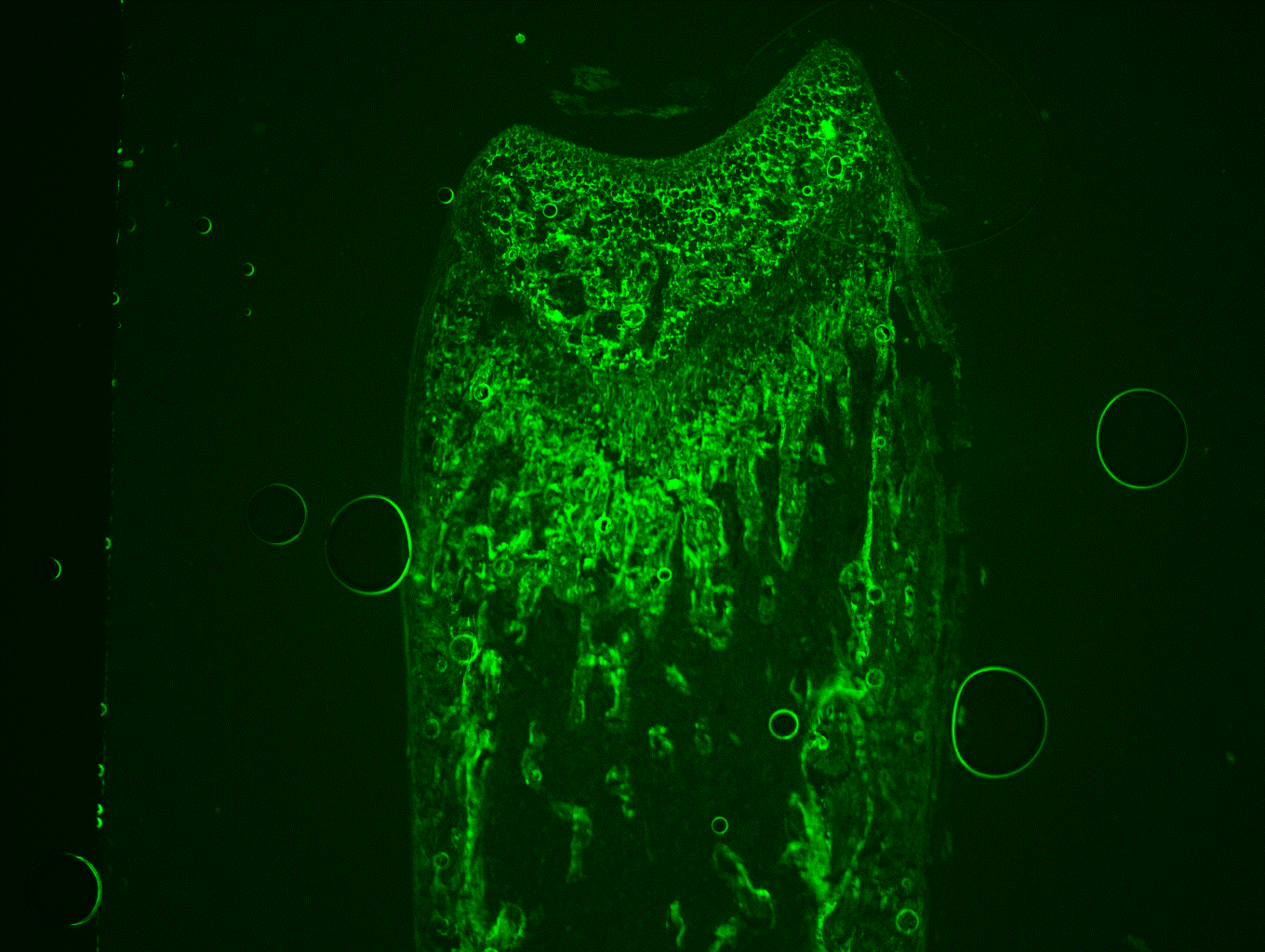

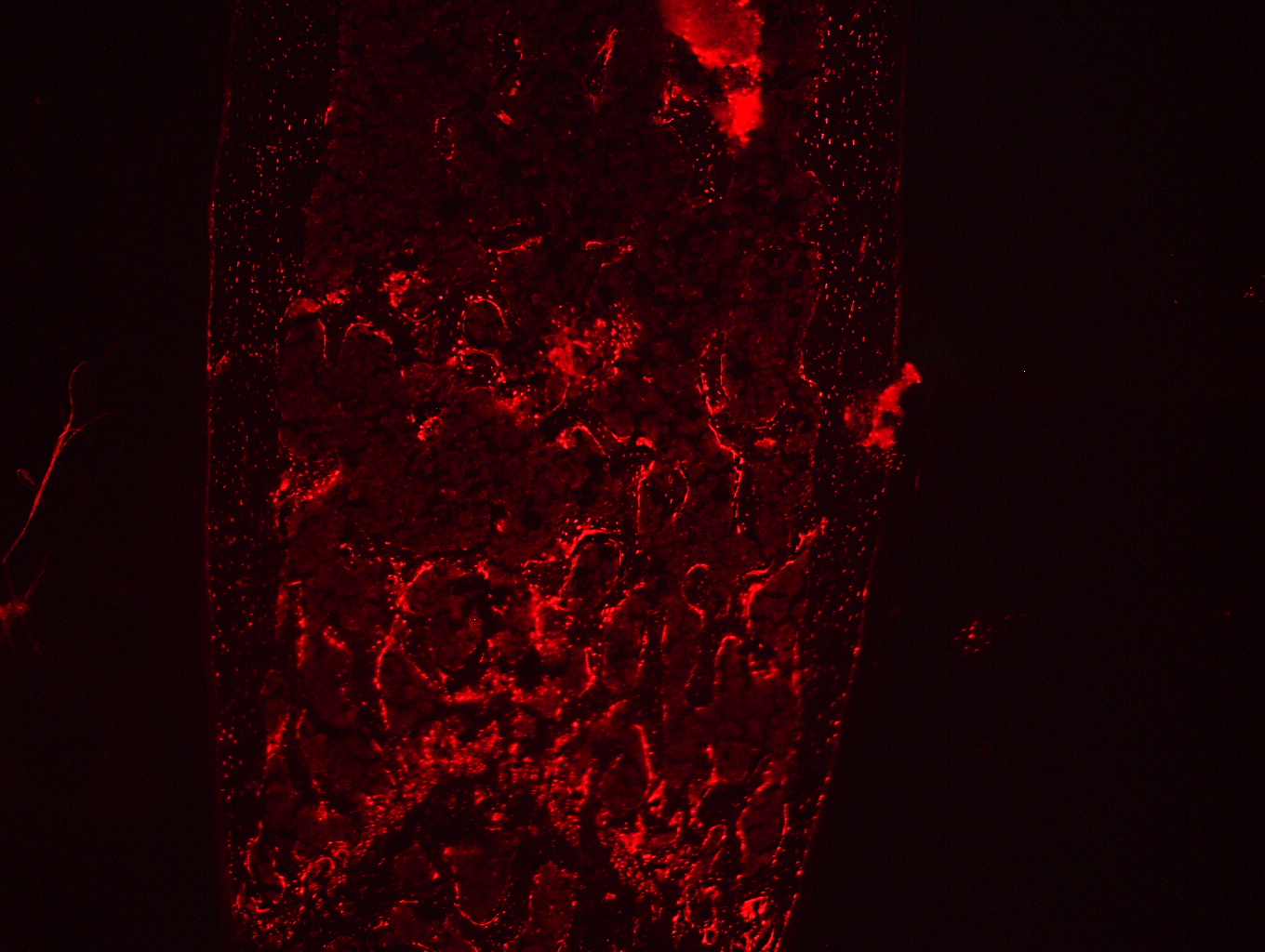


**Prx1-GFP**

**Osterix-mCherry**


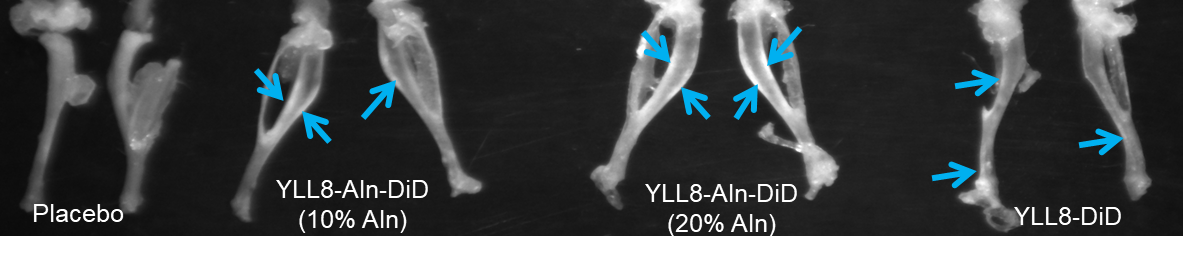
**Supplementary Figure 6.** Near-infrared fluorescence imaging of the tibia after the mice were injected with YLL8-Aln-DiD for 4 hours. Blue arrows indicated bone homing of the injected drug in bright white.

**Table S1. Forty-three amino acids for positions X_1_**

| #1 Fmoc-Orn(Boc)-OH   | #2 Fmoc-HoSer(Trt)-OH   | #3 Fmoc-Acpc-OH   | #4 Fmoc-L-HoCit   |
| --- | --- | --- | --- |
| #5 Fmoc-Hyp(tBu)-OH   | #6 Fmoc-Aad(OtBu)-OH   | #7 Fmoc-D-3-Pal-OH   | #8 Fmoc-L-Phg-OH   |
| #9 Fmoc- Nva-OH   | #10 Fmoc-Dpr(Boc)-OH   | #11 Fmoc-D-Tyr(Me)-OH   | #12 Fmoc- Aib-OH   |
| #13 Fmoc-D-Chg-OH   | #14 Fmoc-4-Apc(Boc)-OH   | #15 Fmoc-Phe(4-Me)-OH   | #16 Fmoc-Nle-OH   |
| #17 Fmoc-D-Phe(3-Cl)-OH   | #18 Fmoc-D-HoPhe-OH   | #19 Fmoc-Aic-OH   | #20 Fmpc-Cha-OH   |
| #21 Fmoc-D-2-Nal-OH   | #22 Fmoc-L-1-Nal-OH   | #23 Fmoc-Phe(3,4-diCl)-OH   | #24 Fmoc-Bpa-OH   |
| #25 Fmoc-D-Ala-OH   | #26 Fmoc-D-Glu(OtBu)-OH   | #27 Fmoc-D-Asn(Trt)-OH   | #28 Fmoc-Gln(Trt)-OH   |
| #29 Fmoc-Ile-OH   | #30 Fmoc-D-Leu-OH   | #31 Fmoc-D-Lys(Boc)-OH   | #32 Fmoc-D-Ser(tBu)-OH   |
| #33 Fmoc-D-Met-OH   | #34 Fmoc-D-Phe-OH   | #35 Fmoc-D-Pro-OH   | #36 Fmoc-Thr(tBu)-OH   |
| #37 Fmoc-Val-OH   | #38 Fmoc-D-Trp(Boc)-OH   | #39 Fmoc-Tyr(tBu)-OH   | $40 Fmoc-Asp(OtBu)-OH   |
| #41 Fmoc-Arg(Pmc)-OH   | #42 Fmoc-D-His(Trt)-OH   | #43 Fmoc-Gly-OH   |  |

**Table S2. Thirty amino acids for position X_2_**

| #1 Fmoc-Ile-OH   | #2 Fmoc-D-Ala-OH   | #3 Fmoc-Abu-OH   | #4 Fmoc-D-Leu-OH   |
| --- | --- | --- | --- |
| #5 Fmoc-D-Pra-OH   | #6 Fmoc-Chg-OH   | #7 Fmoc-Phg-OH   | #8 Fmoc-Nva-OH   |
| #9 Fmoc-Cha-OH   | #10 Fmoc-D-Tyr(tBu)-OH   | #11 Fmoc-Asp(OtBu)-OH   | #12 Fmoc-D-Val-OH   |
| #13 Fmoc-Acpc-OH   | #14 Fmoc-Glu(OtBu)-OH   | #15 Fmoc-Ser(tBu)-OH   | #16 Fmoc-Nle-OH   |
| #17 Fmoc-Bpa-OH   | #18 Fmoc-D-2-Nal-OH   | #19 Fmoc-D-Trp(Boc)-OH   | #20 Fmoc-Ana-OH   |
| #21 Fmoc-HoSer(tBu)-OH   | #22 Fmoc-Ach-OH   | #23 Fmoc-Aad(OtBu)-OH   | #24 Fmoc-D-Thi-OH   |
| #25 Fmoc-Phe(4-Me)-OH   | #26 Fmoc-Aic-OH   | #27 Fmoc-D-Phe-OH   | #28 Fmoc-HoPhe-OH   |
| #29 Fmoc-D-Phe(3-Cl)-OH   | #30 Fmoc-D-Tyr(Me)-OH   |  |  |

**Table S3. Eight amino acids for position X_3_**

| #11 Fmoc-Asp(OtBu)-OH   | #2 Fmoc-Glu(OtBu)-OH   | #3 Fmoc-Aad(OtBu)-OH   | #4 Fmoc-Bmc(OtBu)-OH   |
| --- | --- | --- | --- |
| #5 Fmoc-Ile-OH   | #6 Fmoc-N-Me-Ile-OH   | #7 Fmoc-Leu-OH   | #8 Fmoc-Nle-OH  |

**Table S4. Eighteen building blocks for position X_4_**

| #1 Fmoc-Ile-OH   | #2 Fmoc-Cha-OH   | #3 Fmoc-HoPhe-OH   | #4 Fmoc-Leu-OH   |
| --- | --- | --- | --- |
| #5 Fmoc-Nle-OH  | #6 Fmoc-N-Me-Nle-OH   | #7 Fmoc-N-Me-Ile-OH   | #8 Fmoc-HoArg(Pbf)-OH   |
| #9 Fmoc-Gln(Trt)-OH   | #10 Fmoc-Aup-OH   | #11 Fmoc-Phe(4-CF_3_)-OH   | #12 Fmoc-Cpa-OH   |
| #13 Fmoc-Orn(pyra)-OH   | #14 Fmoc-Phe(3,5-diF)-OH   | #15 Fmoc-HoCit-OH   | #16 Fmoc-Cit-OH   |
| #17 Fmoc-K(A38)-OH  | #18 Fmoc-K(A12)-OH   |  |  |
