# Supplementary material for: Identification of osteogenic progenitor cell-targeted peptides that augment bone formation
Source: Nat Commun. 2020 Aug 27;11:4278. doi: 10.1038/s41467-020-17417-9 (PMC7453024; doi:10.1038/s41467-020-17417-9)
Supplement: Supplementary file 3 — Reporting Summary [file 41467_2020_17417_MOESM3_ESM.pdf]

nature research

Corresponding author(s): Wei Yao

Last updated by author(s): May 3, 2020

Reporting Summary

Nature Research wishes to improve the reproducibility of the work that we publish. This form provides structure for consistency and transparency in reporting. For further information on Nature Research policies, see Authors & Referees and the Editorial Policy Checklist .

Statistics

For all statistical analyses, confirm that the following items are present in the figure legend, table legend, main text, or Methods section.

n/a	Confirmed	
☐	☒	The exact sample size (*n*) for each experimental group/condition, given as a discrete number and unit of measurement
☐	☒	A statement on whether measurements were taken from distinct samples or whether the same sample was measured repeatedly
☐	☒	The statistical test(s) used AND whether they are one- or two-sided<br>*Only common tests should be described solely by name; describe more complex techniques in the Methods section.*
☐	☒	A description of all covariates tested
☐	☒	A description of any assumptions or corrections, such as tests of normality and adjustment for multiple comparisons
☒	☐	A full description of the statistical parameters including central tendency (e.g. means) or other basic estimates (e.g. regression coefficient) AND variation (e.g. standard deviation) or associated estimates of uncertainty (e.g. confidence intervals)
☐	☒	For null hypothesis testing, the test statistic (e.g. *F*, *t*, *r*) with confidence intervals, effect sizes, degrees of freedom and *P* value noted<br>*Give P values as exact values whenever suitable.*
☒	☐	For Bayesian analysis, information on the choice of priors and Markov chain Monte Carlo settings
☒	☐	For hierarchical and complex designs, identification of the appropriate level for tests and full reporting of outcomes
☐	☒	Estimates of effect sizes (e.g. Cohen's *d*, Pearson's *r*), indicating how they were calculated

Our web collection on statistics for biologists contains articles on many of the points above.

Software and code

Policy information about availability of computer code

Data collection	Data were collected from Jan 2013 to Dec. 2019. Data were recorded in the forms of excel data sheets
Data analysis	Prism 8.0 was used for graphing and statistics.

For manuscripts utilizing custom algorithms or software that are central to the research but not yet described in published literature, software must be made available to editors/reviewers. We strongly encourage code deposition in a community repository (e.g. GitHub). See the Nature Research guidelines for submitting code & software for further information.

Data

Policy information about availability of data

All manuscripts must include a data availability statement. This statement should provide the following information, where applicable:

- Accession codes, unique identifiers, or web links for publicly available datasets
- A list of figures that have associated raw data
- A description of any restrictions on data availability

The source data underlying Figs 1a, 2a–d, 6d, h and 7c, and Supplementary Figs 1a and 5d are provided as a Source Data file, and are available from the authors.

Field-specific reporting

Please select the one below that is the best fit for your research. If you are not sure, read the appropriate sections before making your selection.

☒ Life sciences ☐ Behavioural & social sciences ☐ Ecological, evolutionary & environmental sciences

For a reference copy of the document with all sections, see nature.com/documents/nr-reporting-summary-flat.pdf

Life sciences study design

All studies must disclose on these points even when the disclosure is negative.

Sample size	We used our previous data on mice who underwent ovariectomy and received PTH treatment to calculate sample size of n>=5 to achieve power = 0.8, a=0.05 for in vivo studies.
Data exclusions	All the measurements were obtained from distinct samples and not from measuring the same sample repeatedly. All the samples were included in the analysis. All inclusion/exclusion criteria were pre-established, and no animals or samples were excluded from the analysis.
Replication	n=3-11 for in vitro studies and n=5-8 for in vivo studies. All the attempts at replicates were successful for in vitro study.
Randomization	For in vitro studies, the cells were mixed well before they were plated and underwent further differentiation assays. For in vivo studies, mice were randomized into different groups at baseline.
Blinding	investigations were blinded to data analyses

Reporting for specific materials, systems and methods

We require information from authors about some types of materials, experimental systems and methods used in many studies. Here, indicate whether each material, system or method listed is relevant to your study. If you are not sure if a list item applies to your research, read the appropriate section before selecting a response.

Materials & experimental systems

n/a	Involved in the study	
☐	☒	Antibodies
☒	☐	Eukaryotic cell lines
☒	☐	Palaeontology
☐	☒	Animals and other organisms
☒	☐	Human research participants
☒	☐	Clinical data

Methods

n/a	Involved in the study	
☒	☐	ChIP-seq
☒	☐	Flow cytometry
☒	☐	MRI-based neuroimaging

Antibodies

Antibodies used	Akt antibody: ab179463, 1:10,000; p-Akt antibody: ab 81283, 1:5000; b-actin antibody, ab8229, 1:500; goat-anti-rabbit IgG H&L (HRP), ab205718, 1:10000.
Validation	per manufacturer's recommendations for western blot, user's reviews and validated by pilot runs

Animals and other organisms

Policy information about studies involving animals; ARRIVE guidelines recommended for reporting animal research

Laboratory animals	We used 2 and 4-monthold C57BL/6 mice, both female and males, 2-monthold male Prx1-CreERT-GFP, 2-month-old female of 129/Svj background in this report. All the animal protocols were approved by UC Davis IACUC
Wild animals	no wild animals were used in the study.
Field-collected samples	o field collected samples were used in the study.
Ethics oversight	The Institutional Animal Care and Use Committee at The University of California, Davis, committees that approved the protocol,

Note that full information on the approval of the study protocol must also be provided in the manuscript.
